# Documented Paternity Despite Azoospermia Post-vasectomy

**DOI:** 10.7759/cureus.72619

**Published:** 2024-10-29

**Authors:** Teresa Bernardes, Ting Y Wu, Christine C Greves, Steve Carlan

**Affiliations:** 1 Internal Medicine, Orlando Regional Medical Center, Orlando, USA; 2 Obstetrics and Gynecology, Orlando Regional Medical Center, Orlando, USA; 3 Obstetrics, Orlando Regional Medical Center, Orlando, USA

**Keywords:** paternity, paternity testing, polymerase chain reaction arrangement, semen analysis, vasectomy

## Abstract

Vasectomy is a permanent and safe method of male contraception. One commonly used definition of success is azoospermia in one or two semen analyses after 20 ejaculates after three months. Failure is higher if less than 1.0 cm of vas deferens is removed or if a postsurgical infection occurs. There can be obvious social and legal consequences following a failed vasectomy and subsequent pregnancy. Paternity testing of the prospective father and child using automated DNA investigation employing amplified polymerase chain reaction (PCR) to compare short tandem repeat (STR) loci is accurate and readily available.

A 32-year-old healthy male presented for elective sterilization after his wife delivered her third healthy full-term infant. The vasectomy was performed by ligation and excision as an outpatient. There were no intraoperative or postoperative complications. He had one semen analysis 48 days post-vasectomy that confirmed azoospermia. His wife conceived 119 days post-vasectomy, and he had two subsequent negative sperm counts. She delivered a full-term infant 385 days post-vasectomy. DNA paternity testing confirmed he was the father.

Either the semen analysis was incorrect because of human error during the sperm count or small numbers of viable sperm were present despite reported preconception azoospermia. Human error is unlikely since all three tests were negative. This early failure probably does not represent recanalization but the transient release of sperm stored in the seminal vesicles or vas deferens. The time from vasectomy to laboratory-confirmed azoospermia varies. The time from vasectomy to functional azoospermia is not known, which is the reason for counseling about the risks of rare unexpected failures despite laboratory-confirmed azoospermia.

## Introduction

Vasectomy is a widely accepted and highly effective method of male contraception that has been performed nationwide since the mid-20th century [1]. In the United States, 80% of the procedures are performed by outpatients and around 20% by non-urologists [2]. The procedure involves surgical transection or occlusion of the right and left vas deferens to prevent the passage of sperm into the ejaculate. It is considered by many as the definitive irreversible male sterilization method when measured by safety, cost, and efficacy [3]. After a vasectomy, male sterility is confirmed by obstructive azoospermia or the complete absence of viable or motile sperm in the semen through semen analysis conducted at intervals post-operatively. Some data suggest the patient may stop using contraception post-vasectomy before three months or 20 ejaculations if they are azoospermic on sperm count [4]. However, by convention, patients are generally considered sterile after one or two consecutive azoospermic samples or have met criteria of less than 100,000 non-motile spermatozoa/mL in a single ejaculate sample taken three months after the procedure [5]. Generally, 20 ejaculates are considered necessary to ensure a certified negative test. Despite this high level of effectiveness, cases of post-vasectomy pregnancies, though rare, have been documented in the medical literature [6]. The risk of pregnancy following a vasectomy with negative semen analysis is estimated to be between one in every 250 to 2000 patients [6]. These cases of vasectomy failure are usually associated with recanalization and confirmed presence of sperm in the new semen analysis. Spontaneous reanastomosis of the separated ends is most likely if a surgical abscess occurs or a smaller (<1 cm) segment of vas deferens is resected [7].

Paternity testing by polymerase chain reaction (PCR)-based short tandem repeat marker (STR) assessment can result in a probability of paternity >99.999% [8]. We present a case of confirmed paternity by DNA testing after histologically confirmed successful vasectomy and three negative ejaculate samples resulting in a full-term vaginal birth after cesarean delivery.

## Case presentation

**Figure 1:**
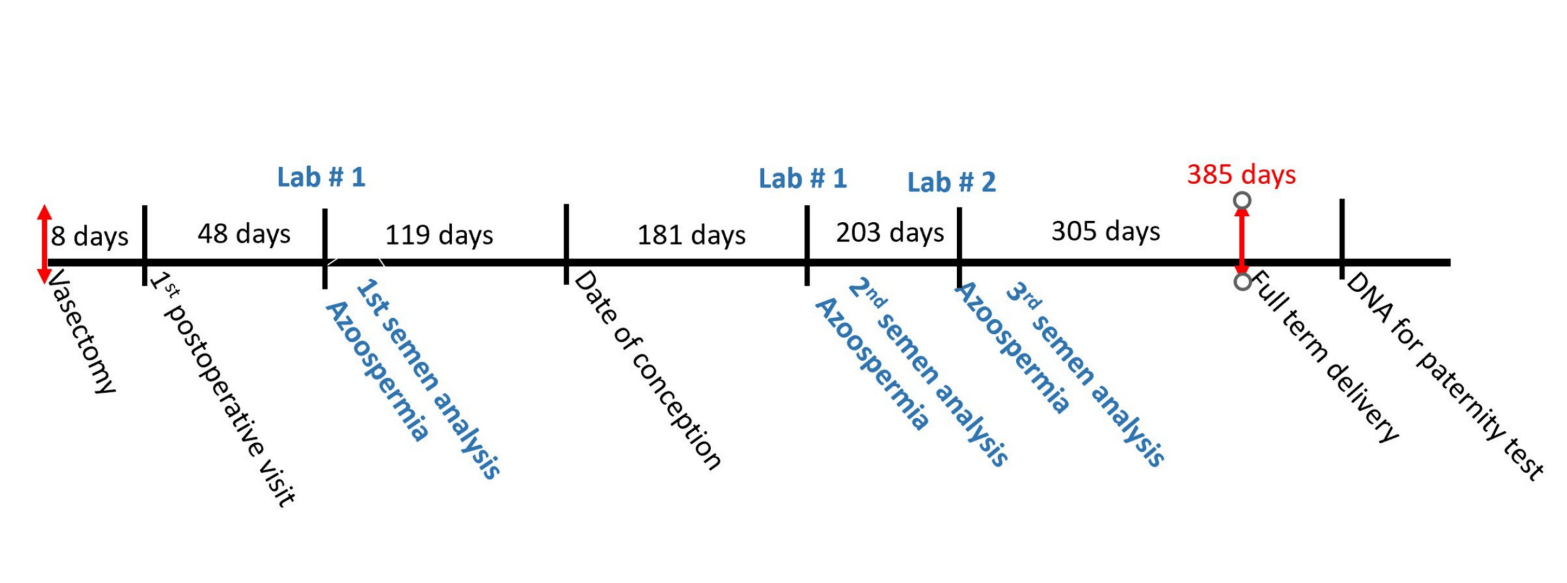
Timeline over 385 days showing the days of semen analysis in blue text and the date of delivery in red text.

A healthy 34-year-old G4P4004 female had an unexpected pregnancy after her third child. Her previous pregnancies had been uncomplicated vaginal deliveries, followed by a cesarean delivery of her third child because of persistent breech presentation at term in labor. The current delivery was a successful vaginal birth after cesarean at term. Her 32-year-old healthy husband had undergone an elective vasectomy for surgical sterilization approximately one year before this last delivery. The history of his vasectomy revealed that under local anesthesia, a board-certified, experienced urologist removed 1.1 cm segments of both the left and right vas deferens through an incision in the right and left hemiscrotum. There were no intraoperative or postoperative complications. The patient returned for his postoperative visit in one week without complaints. The pathology report confirmed vas deferens segments, 1.1 cm on both the right and left. His first post-vasectomy semen analysis was 48 days after the procedure and confirmed azoospermia. The number of ejaculates before this semen analysis is unknown. He did not return for his second semen analysis until his wife became pregnant. His second semen analysis was 181 days after the vasectomy and also confirmed azoospermia (Figure 1).

Of note based on clinical findings, his wife conceived 119 days after the vasectomy and the husband’s request for the second semen analysis reflected his effort to collect information to establish paternity. He had failed to follow up with the urologist’s office for his scheduled second post-vasectomy semen analysis. Seeking further clarification, the couple consulted a second urologist, and repeat semen analyses confirmed persistent azoospermia. This third negative semen analysis occurred 203 days after the vasectomy and he inquired about definitive DNA paternity testing at the time. Given questions regarding paternity, the family was advised to obtain a DNA analysis of the child, which was obtained after the birth of the infant. A PCR-based STR assessment of the child and alleged father (the husband) revealed the probability of paternity >99.999%. The mother chose to have an intrauterine contraceptive device inserted for contraception and their matrimonial union is stable.

## Discussion

The finding of pregnancy in this context is particularly unusual, as it occurred despite confirmatory semen analyses showing obstructive azoospermia on days 48, 181, and 203 post-vasectomy in two different labs. The patient conceived 119 days post-vasectomy, despite her partner not obtaining the standard post-90-day azoospermia test results, and having an unknown number of ejaculations since vasectomy. However, he did have one preconception negative test and two negative tests after the pregnancy was confirmed. Edwards demonstrated that since the time to obstructive azoospermia is so highly variable discontinuing contraception is reasonable after a sperm count revealed no motile sperm is confirmed at any time [4]. In addition, Barone demonstrated that using the current definition for sterilization success, 11.5% of all subjects would have failed their vasectomies since the time required to reach azoospermia and the number of ejaculates widely varies [9]. Barone defined success as a negative sperm count, not pregnancy prevention. That is a critical component in patient counseling. Although extremely rare, as this case shows, one can be technically sterile but functionally fertile after a post-vasectomy negative sperm count, and it may not be secondary to recanalization of the vas deferens. There remains controversy in the role ejaculation number plays in both post-vasectomy sperm count and the overall length of time to full sterility.

Considering the positive paternity in our case, only four possibilities could explain the unexpected conception. Recanalization, lab error, spontaneous intermittent release of sperm stored in the proximal genitourinary system, or malfeasance by intentional sperm storage and reinjection through artificial insemination with the husband’s stored sperm [10]. First, given that the patient had documented azoospermia post-operatively and also after the pregnancy was confirmed, the probability of spontaneous recanalization of the vas deferens is exceedingly low even though the number of ejaculates after the procedure was unknown [9]. Spontaneous recanalization, where the severed ends of the vas deferens reconnect and allow sperm to pass through, is a known but rare complication of vasectomy [6]. Greater than 1 cm of vas was ligated and removed, and there were no postoperative surgical site infections, so spontaneous recanalization probably did not occur. Second, another possibility could be human error in the lab. Inspecting counting chambers for motile or nonmotile sperm is tedious and potentially the sperm could be missed, but two separate labs on three separate occasions reported azoospermia. Third, a more likely explanation could involve the intermittent or transient presence of sperm that was not captured during routine semen analysis. Although rare, there have been reports of cases where sperm appears intermittently in the ejaculate after vasectomy due to possible micro-recanalization or the presence of residual sperm stored in the vas deferens or seminal vesicles, which are released intermittently [7,11-13]. This would seem to be highly dependent on ejaculation frequency. Fourth and least likely is the intentional reintroduction of autologous cryo-preserved or stored sperm for nefarious purposes and personal agendas. There were no reported legal actions and the couple requested a paternity test so this cause is unlikely. It is unlikely that operative technique was a variable in this outcome. A urologist with experience performed the case, and histopathology was certified. Also, vasectomy, in general, is not a difficult procedure. Finally, there may be other less understood mechanisms that could account for the presence of viable sperm despite the confirmed azoospermia and paternity.

Considering the documented paternity, sperm must have been present on conception day 119 after the vasectomy but not seen in the counting chamber on day 48. This report is consistent with Barone’s study that analyzed serial sperm counts following vasectomy. He concluded that developing standardized guidelines for sperm clearance is difficult using either time or number of ejaculations [9]. His study, however, did not include any post-vasectomy pregnant women, and this missing factor plus our DNA-documented paternity makes our case baffling. 

Paternity testing is safe, reproducible, rapid, and only requires a small amount of DNA. Since the 1990s, PCR has become the method of choice. A paternity PCR method is used to target STR in selected regions of DNA and a comparison is made to determine the genetic relationship among those tested [8].

The testing in this case allowed certainty of fatherhood but not the reason for the failure to achieve unwanted fertility. In all likelihood, this unexpected pregnancy represents a case of the presence of residual sperm stored in the vas deferens or seminal vesicle and released during coitus 119 days after the vasectomy, which was 71 days after the documented azoospermia [11,12]. The length of time that sperm cycles, develops, and lives in the testis before being reabsorbed varies but has been reported to be 70 days [14]. Human males continue to manufacture sperm despite the vasectomy so this cycle is probably unimportant in a proven fertile post-vasectomy male without recanalization.

This case reinforces the importance of counseling patients about the limitations and rare risks associated with vasectomy, even in the context of confirmed post-vasectomy azoospermia. Our patient’s preoperative counseling was standard and included data describing a failure rate of one in 250 and a late failure from recanalization of one in 2,000 [6].

One other item that could be covered during counseling about post-vasectomy failures is that there are no data defining the risk of another pregnancy for a couple whose male partner has post-vasectomy azoospermia and fathers a subsequent live birth. The best option is probably to suggest another form of contraception. This case emphasizes the need for further research into the underlying mechanisms that could contribute to vasectomy failure in seemingly impossible scenarios.

It has been shown that 36-45% of men in the United States do not return for the recommended timed semen analyses [15]. While the likelihood of failure is extremely low, it is not zero, and patients should be made aware of the remote possibility of pregnancy. Genetic testing in this instance confirmed paternity with a probability of more than 99.9999%, effectively excluding non-paternity scenarios and confirming that the vasectomy had failed.

Whether all surgically removed segments of the vas deferens should be assessed histologically remains controversial but there is a clear benefit to providing feedback to the surgeon [16]. It is increasingly clear that thorough preoperative counseling can be important to mitigate the legal, social, and emotional consequences of a failed vasectomy [17]. Multiple reports confirm that there are no documented long-term health risks to vasectomy, but there is general agreement more research is needed to investigate the role of transient sperm release, refining semen analysis techniques, and studying long-term outcomes of vasectomy patients [3].

## Conclusions

In conclusion, what makes this case unique is the occurrence of pregnancy after documented azoospermia before and after conception after a vasectomy that removed >1 cm of vas deferens bilaterally. Two separate labs with three separate sperm counts concluded he was azoospermic. DNA testing proved fatherhood, histologic examination confirmed a successful procedure. This case highlights a rare instance of pregnancy following vasectomy despite confirmed obstructive azoospermia, suggesting that unknown or less common mechanisms may sometimes contribute to such outcomes. It highlights the importance of comprehensive patient counseling, consideration of rare complications, and ongoing research into the pathophysiology of vasectomy failure. Importantly, this conception occurred 119 days after vasectomy, which may have implications for future patient counseling.
